# Detection of chronic brain damage by diffusion-weighted imaging with multiple *b* values in patients with type 2 diabetes

**DOI:** 10.1097/MD.0000000000004726

**Published:** 2016-09-02

**Authors:** Tieli Liu, Yunpeng Han, Lemei Tang, Jianlin Wu, Yanwei Miao, Bingbing Gao, Jin Shang

**Affiliations:** a Department of Radiology, First Affiliated Hospital; b Graduate School, Dalian Medical University; c Department of Radiology, Dalian Municipal Third People's Hospital; d Department of Radiology, Affiliated Zhongshan Hospital, Dalian University, Liaoning Province, China.

**Keywords:** chronic brain damage, diffusion-weighted imaging, multiple *b* values, type 2 diabetes

## Abstract

The aim of the study was to evaluate the performance of parameters obtained from diffusion-weighted imaging (DWI) with multiple *b* values in the detection of chronic brain damage in patients with type 2 diabetes.

We enrolled 30 patients with or without abnormalities on brain magnetic resonance imaging (lacunar infarction, leukoaraiosis, and/or brain atrophy) and 15 nondiabetic controls; obtained DWI parameters that included apparent diffusion coefficient (ADC), fast ADC (ADC_fast_), slow ADC (ADC_slow_), fraction of fast ADC (*f*), distributed diffusion coefficient (DDC), and stretched exponential (α); and performed receiver operating characteristic (ROC) analysis to evaluate the performance of parameters for the detection of chronic brain damage.

The parameters ADC, ADC_slow_, *f*, and DDC were increased, whereas parameters ADC_fast_ and α were decreased in type 2 diabetes patients compared with controls without diabetes. The centrum semiovale showed the most significant change in the evaluated parameters, and the changes in parameters ADC_slow_, *f*, and DDC were greater than the changes in other parameters. There was no significance between parameters of the biexponential model (ADC_fast_, ADC_slow_, *f*) and parameters of the stretched model (DDC, α), but parameters of both these models were superior to the parameter of monoexponential model (ADC). Moreover, ROC analysis showed that ADC_slow_ of the centrum semiovale supplied by the anterior cerebral artery had the highest performance for detection of chronic brain damage (area under the ROC curve of 0.987, 93.3% sensitivity, and 100% specificity).

Our study shows that DWI with multiple *b* values can quantitatively access chronic brain damage and may be used for detection and monitoring in type 2 diabetes patients.

## 1 Introduction

The International Diabetes Federation (IDF) data show that 415 million adults (1 in 11 adults) are suffering from diabetes worldwide, including 109.6 million adults in China. By 2040, 642 million adults (1 in 10 adults) will have diabetes worldwide.^[1]^ Patients with type 2 diabetes have a greatly increased risk of cardiovascular disease and microvascular disease, including chronic brain damage. It has been shown that 19.8–44.9% of type 2 diabetes patients have chronic brain damage,^[2]^ which can lead to lacunar infarction, leukoaraiosis, and brain atrophy, as well as cognitive deficits and neurophysiological changes.^[3]^ The development of chronic brain damage is associated with atherosclerosis, chronic ischemia, small vascular disease (SVD), oxidative stress, and blood–brain barrier dysfunction.^[4–9]^

Diffusion-weighted imaging (DWI), a form of magnetic resonance imaging (MRI), is a valuable noninvasive technique that plays an important role in the diagnosis of ischemic stroke, especially super-acute or acute cerebral infarction.^[10]^ DWI is sensitive to molecular diffusion, which is the thermally induced motion of water molecules in biological tissues, called Brownian motion. Most of DWI is performed using a monoexponential model of diffusion signal decay, and an apparent diffusion coefficient (ADC) value is obtained. However, DWI decay in the brain does not follow the monoexponential model, and an ADC value may not be able to reflect water diffusion in the brain accurately. The intravoxel incoherent motion (IVIM) theory has been developed to separate the pure water diffusion and the microcirculation perfusion of tissues using the biexponential model,^[11]^ and the stretched exponential model has been developed to describe diffusion-related signal decay as a continuous distribution of sources decaying at different rates. As there is no assumptions made about the number of participating sources, the stretched exponential model can reflect the heterogeneity within the voxel.^[12]^ Parameters of the biexponential model include standard ADC, fast ADC (ADC_fast_), slow ADC (ADC_slow_), and fraction of fast ADC (*f*), whereas parameters of the stretched exponential model include distributed diffusion coefficient (DDC), and stretched exponential (α). There are at least 2 different diffusion rates (fast and slow) that are associated with the decay of water signal in the human brain.^[13]^ Due to this biexponential behavior in brain, DWI with multiple *b* values, which is based on a biexponential model^[11,14]^ and/or a stretched exponential model without assumptions made,^[12]^ has been used in ischemic stroke and brain tumors.^[15–17]^

To our knowledge, application of DWI with multiple *b* values in the detection of chronic brain damage in type 2 diabetic patients has not been investigated. Thus, in the present study, we evaluated the performance of parameters obtained from DWI with multiple *b* values, using monoexponential, biexponential, and stretched exponential models, in the detection of chronic brain damage in patients with type 2 diabetes.

## 2 Methods

### 2.1 Subjects

From February 2014 to March 2015 at First Affiliated Hospital of Dalian Medical University, we enrolled 45 subjects who included 30 patients with type 2 diabetes and 15 controls without diabetes. The diagnosis of type 2 diabetes was made according to the American Diabetes Association guidelines (2012). Those who had a history of brain surgery, brain tumor, cerebrovascular disease, or other diseases of the central nervous system were excluded from the study. The enrolled subjects in the 3 groups were balanced with respect to gender and age. The 15 nondiabetic controls had a mean ± SD age of 60.43 ± 2.61 years (range, 57–66) years; 7 were women. The 15 diabetes patients whose brain MRI showed no abnormalities (MRI (–) group) had a mean ± SD age of 60.67 ± 1.67 years (range, 57–65 years) and a mean history of type 2 diabetes of 6.4 ± 3.87 years (range, 1–15 years); 8 were women. The 15 diabetic patients whose brain MRI showed lacunar infarction, leukoaraiosis, and/or brain atrophy (MRI (+) group) had a mean ± SD age of 61.17 ± 1.13 years (range, 59–66 years) and a mean history of type 2 diabetes of 10.47 ± 5.59 years (range, 3–22 years); 9 were women. All the subjects were right-handed.

The study was approved by the Medical Ethics Committee of First Affiliated Hospital of Dalian Medical University (LCKY2014-47) and performed in accordance with the ethical guidelines of the Declaration of Helsinki. Informed consent was obtained from each subject.

### 2.2 Image acquisition

MRI scans of the brain were obtained with use of a 1.5-Tesla scaner (GE Healthcare) with an 8-channel phased-array head coil. The image protocol included sagittal T1-weighted imaging (T1WI), axial T1WI, T2-weighted imaging (T2WI), axial T2 fluid-attenuated inversion recovery (FLAIR), and DWI. DWI scans were obtained with the following parameters: TR = 3400 ms, TE = 102 ms, slice thickness = 6 mm, interslice gap = 1 mm, FOV = 23.0 cm × 20.8 cm, matrix of 192 × 192; and with 11 *b* values (0, 100, 200, 400, 600, 800, 1000, 1500, 2000, 2500, and 3000 s/mm^2^). The DWI acquisition time was 5 minutes 28 seconds.

### 2.3 Image analysis

**Figure 1:**
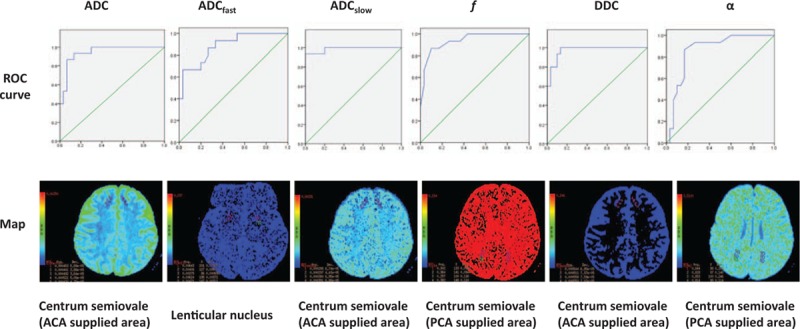
Maps and measurements of parameters of diffusion-weighted imaging with multiple *b* values and receiver operating characteristic (ROC) analysis. Maps and measurement of parameters (ADC, ADC_fast_, ADC_slow_, *f*, DDC and α) in the anterior limb of the internal capsule, posterior limb of the internalcapsule, lenticular nucleus, and centrum semiovale that is supplied by ACA, MCA, and PCA. Receiver operating characteristic (ROC) analysis was performed to determine the area under the ROC curve (AUC) of each individual parameter for the detection of chronic brain damage in patients with type 2 diabetes. The upper panel shows ROC curves for parameters of diffusion-weighted imaging with multiple *b* values, and the lower panel shows maps in a 62-year-old woman with type 2 diabetes. The AUCs for the corresponding ROC curves are 0.944 (ADC), 0.886 (ADC_fast_), 0.987 (ADC_slow_), 0.938 (*f*), 0.938 (DDC), 0.860 (α). ADC = apparent diffusion coefficient, ADC_fast_ = fast ADC, ADC_slow_ = slow ADC, *f* = fraction of fast ADC, DDC = distributed diffusion coefficient, α = stretched exponential, ACA = anterior cerebral artery, AUC = areas under the receiver-operating characteristic curve, MCA = middle cerebral artery, PCA = posterior cerebral artery, ROC = receiver operating characteristic.

Image analysis was performed automatically by the workstation (Advantage Workstation 4.4, GE Healthcare) with the use of the multi-ADC analysis algorithm (MADC) software in the Functool software package (GE Healthcare). Maps of standard ADC, fast ADC (ADC_fast_), slow ADC (ADC_slow_), fraction of fast ADC (*f*), distributed diffusion coefficient (DDC), and stretched exponential (α) were obtained. Parameters of ADC, ADC_fast_, ADC_slow_, *f*, DDC, and α were measured in the anterior limb of the internal capsule, posterior limb of the internal capsule, lenticular nucleus, and centrum semiovale that is supplied by anterior cerebral artery (ACA), middle cerebral artery (MCA), and posterior cerebral artery (PCA). To avoid the influence of the blood vessel, cerebrospinal fluid, and infarction, we drew a small region of interest (ROI, 20–40 pixels). The ROIs analysis on the parametric maps was performed by 2 radiologists who had 11 and 13 years of MRI diagnosis experience, respectively. The ROIs was drawn for 3 times at each site, and the mean of each measurement was used for analysis (Fig. 1).

### 2.4 Statistical analysis

Concordance between different radiologists was quantified using intraclass correlation coefficients (ICC, ICC of 0.9). The paired *t*-test was used to compare the parameters between the left and the right at each site, and the means of parameters were calculated if there was no significance between 2 sides. Analysis of variance (ANOVA) was used to compare the parameters at each site in the 3 groups. Receiver-operating characteristic (ROC) analysis was performed to determine the area under the ROC curve (AUC) of each individual parameter for the detection of chronic brain damage in patients with type 2 diabetes. Data are expressed as means and standard deviation (SD), and a *P* value of <0.05 was considered indicative of statistical significance. Analyses were carried out using the statistical software package software package (SPSS, Version 17.0, SPSS Inc, Chicago, IL).

## 3 Results

### 3.1 Monoexponential model (ADC)

In the monoexponential model, ADC is used to quantitate water diffusion. We found that ADC was significantly increased in the 2 groups of patients with type 2 diabetes in the anterior limb of the internal capsule and centrum semiovale supplied by the ACA, as compared with the control group (*P* < 0.05; Table 1). Of the patients with type 2 diabetes, ADC was significantly higher in the MRI (+) group than in the MRI (–) group, in the posterior limb of the internal capsule, and in the centrum semiovale supplied by the ACA, MCA, and PCA (*P* < 0.05).

**Table 1:**
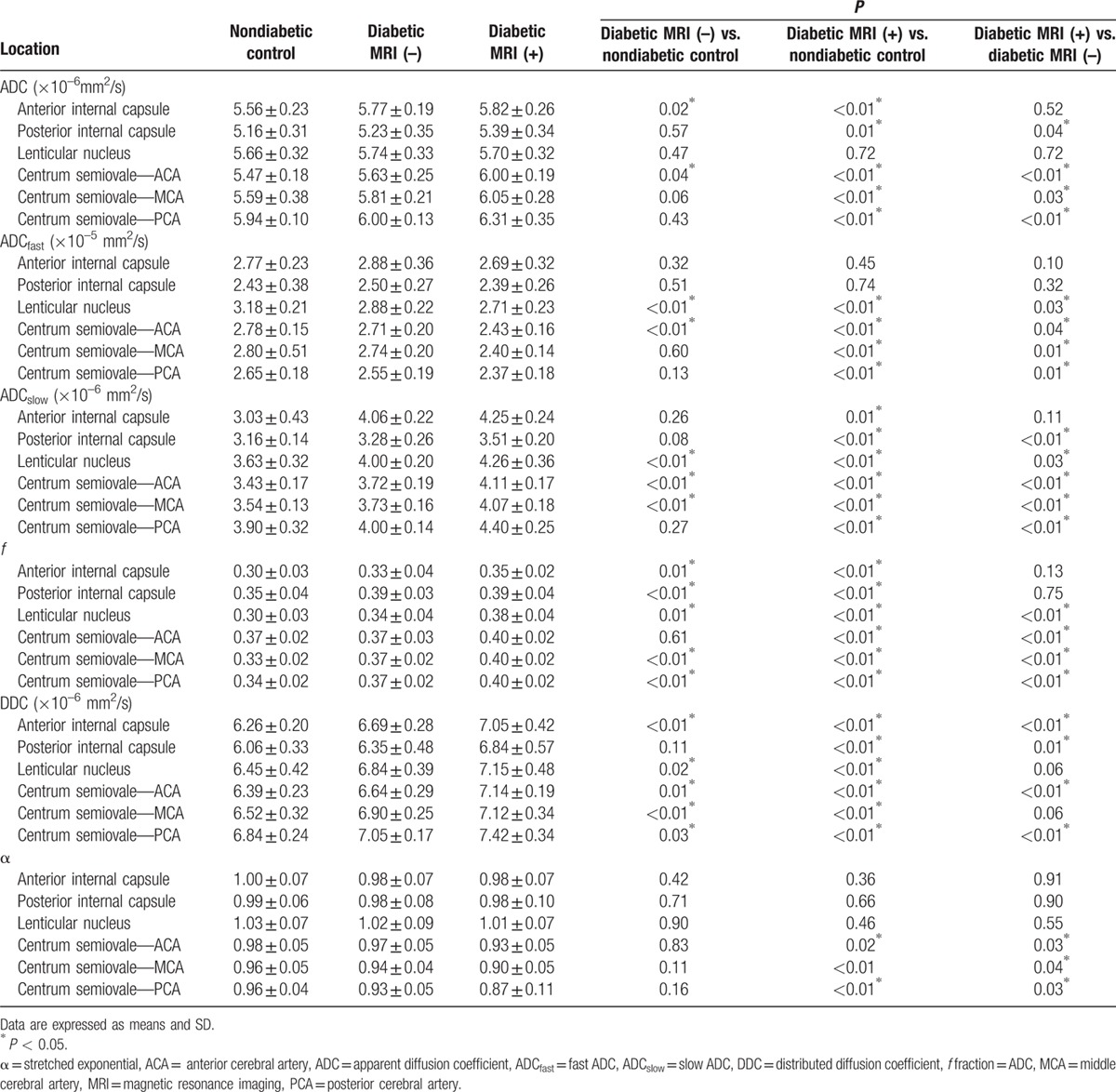
Parameters of diffusion-weighted imaging with multiple *b* values in subjects.

### 3.2 Biexponential model (ADC_fast_, ADC_slow_, and *f*)

In the biexponential model, ADC_fast_ was significantly decreased in the 2 groups of patients with type 2 diabetes in the lenticular nucleus and centrum semiovale supplied by ACA, as compared with the control group (*P* < 0.05; Table 2). Of the patients with type 2 diabetes, ADC_fast_ was significantly lower in the MRI (+) group than in the MRI (–) group and in the lenticular nucleus and centrum semiovale supplied by ACA, MCA, and PCA (*P* < 0.05).

**Table 2:**
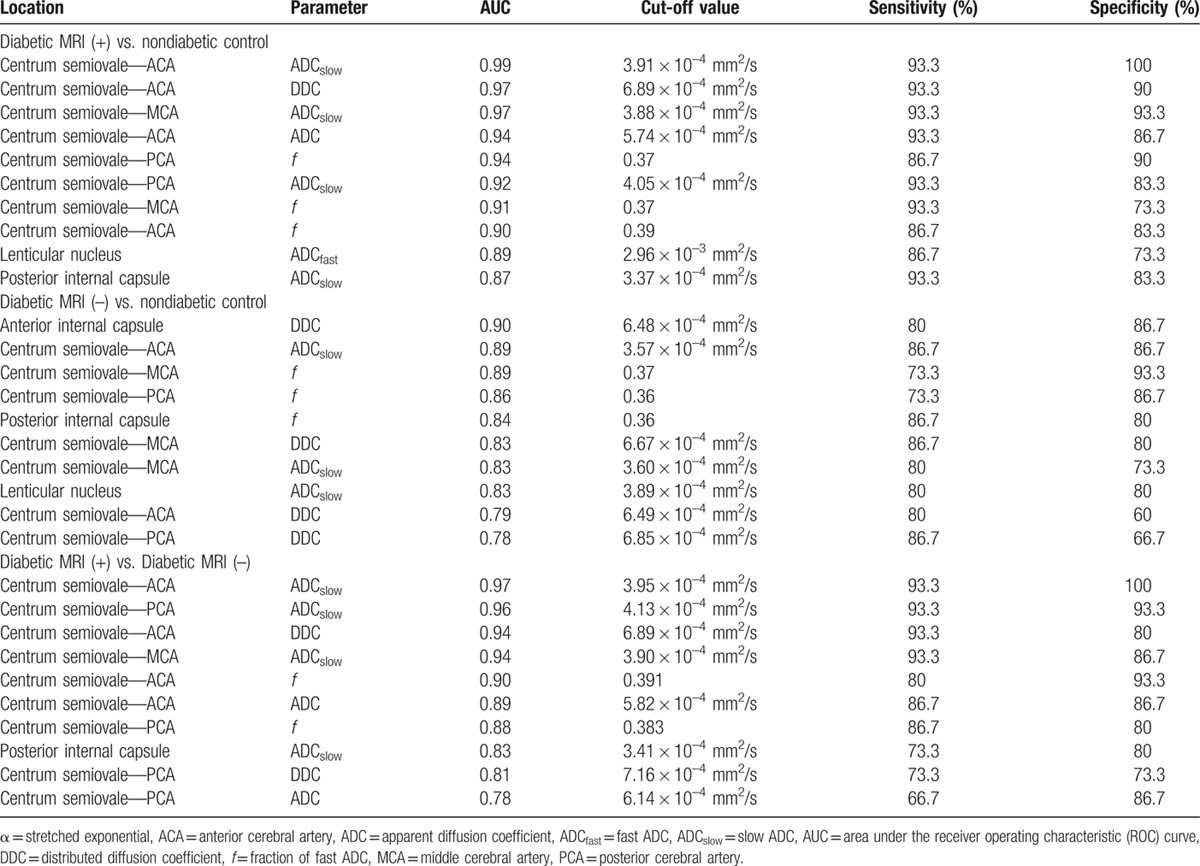
Performance of parameters for the detection of chronic brain damage (top 10 for each comparison).

ADC_slow_ was significantly increased in the 2 groups of patients with type 2 diabetes in the lenticular nucleus and centrum semiovale supplied by the ACA and MCA, as compared with the control group (*P* < 0.05; Table 2). Of the patients with type 2 diabetes, ADC_slow_ was significantly higher in the MRI (+) group than in the MRI (–) group, in the posterior limb of the internal capsule, and in the lenticular nucleus and centrum semiovale supplied by ACA, MCA and PCA (*P* < 0.05).

*f* was significantly increased in the 2 groups of patients with type 2 diabetes in the anterior limb of the internal capsule, posterior limb of the internal capsule, lenticular nucleus, and centrum semiovale supplied by the MCA and PCA, as compared with the control group (*P* < 0.05; Table 2). Of the patients with type 2 diabetes, *f* was significantly higher in the MRI (+) group than in the MRI (–) group and in the lenticular nucleus and centrum semiovale supplied by ACA, MCA, and PCA (*P* < 0.05).

### 3.3 Stretched exponential model (DDC, α)

In the stretched exponential model, DDC was significantly increased in the 2 groups of patients with type 2 diabetes in the anterior limb of the internal capsule, lenticular nucleus, and centrum semiovale supplied by the ACA, MCA and PCA, as compared with the control group (*P* < 0.05; Table 2). Of the patients with type 2 diabetes, DDC was significantly higher in the MRI (+) group than in the MRI (–) group, in the anterior limb of the internal capsule, posterior limb of the internal capsule, and centrum semiovale supplied by ACA and PCA (*P* < 0.05).

α was significantly higher in the MRI (+) group than in the control group and the MRI (–) group, specifically in the centrum semiovale supplied by the ACA, MCA, and PCA (*P* < 0.05; Table 2). In contrast, there was no significance between the MRI (–) type 2 diabetes group and the control group.

### 3.4 ROC analysis

In the monoexponential model, ROC analysis showed that ADC yielded an AUC of 0.94 in the centrum semiovale supplied by ACA (Fig. 1). A cutoff value of 5.76 × 10^–4^ mm^2^/s maximized the combined sensitivity and specificity (Youden's index),^[18]^ and this parameter has 93.3% sensitivity and 86.7% specificity to discriminate between the MRI (+) group and the control group (Table 2).

In the biexponential model, ROC analysis showed: (1) ADC_fast_ yielded an AUC of 0.89 in the lenticular nucleus (Fig. 1). With the cutoff value of 2.96 × 10^–3^ mm^2^/s, ADC_fast_ has 86.7% sensitivity and 73.3% specificity to discriminate between the MRI (+) group and control group (Table 2). (2) ADC_slow_ yielded an AUC of 0.99 in the centrum semiovale supplied by ACA (Fig. 1). With the cutoff value of 3.91 × 10^–4^ mm^2^/s, ADC_slow_ has 93.3% sensitivity and 100% specificity to discriminate between the MRI (+) group and control group (Table 2). (3) *f* yielded an AUC of 0.94 in the centrum semiovale supplied by PCA (Fig. 1). With the cutoff value of 0.37, *f* has 86.7% sensitivity and 90% specificity to discriminate between the MRI (+) group and control group (Table 2).

In the stretched exponential model, ROC analysis showed: (1) DDC yielded an AUC of 0.94 in the centrum semiovale supplied by ACA (Fig. 1). With the cutoff value of 6.89 × 10^–4^ mm^2^/s, DDC has 93.3% sensitivity and 90% specificity to discriminate between the MRI (+) group and control group (Table 2). (2) α yielded an AUC of 0.86 in the centrum semiovale supplied by the PCA (Fig. 1). With the cutoff value of 0.93, α has 76.0% sensitivity and 83.3% specificity to discriminate between the MRI (+) group and control group (Table 2).

### 3.5 Performance of parameters for the detection of chronic brain damage

On the basis of the AUC, sensitivity, and specificity, we evaluated the performance of each individual parameter for the detection of chronic brain damage in patients with type 2 diabetes. We found that parameters of biexponential and stretched models were superior to the parameter of monoexponential model (ADC) at each site (Table 2), but there was no significance between parameters of the biexponential model and those of the stretched model. Moreover, we found that ADC_slow_ of the centrum semiovale supplied by the ACA had the highest performance to discriminate either between the MRI (+) group and control group (AUC of 0.99, 93.3% sensitivity and 100% specificity), or between the MRI (+) group and MRI (–) group (AUC of 0.97, 93.3% sensitivity, and 100% specificity).

## 4 Discussion

In the present study, we evaluated the performance of parameters obtained via DWI with multiple *b* values in the detection of chronic brain damage in type 2 diabetes patients. We found that DWI parameters of biexponential and stretched exponential models were superior to the parameter of monoexponential model (ADC) and that ADC_slow_ of the centrum semiovale supplied by the ACA had the highest performance compared that of other parameters. Our study suggests that parameters of DWI with multiple *b* values can be used to quantitatively evaluate chronic brain damage in type 2 diabetes and that parameters of the biexponential model and stretched-exponential model are more sensitive and more accurate than parameters of the monoexponential model.

A biexponential model has been proposed to describe the diffusion and the consequent signal decay in DWI.^[13,19–21]^ Our results showed that parameters of biexponential were superior to the parameter of monoexponential model for the detection of chronic brain damage in type 2 diabetic patients. It has been demonstrated that a fast ADC (ADC_fast_) and a slow ADC (ADC_slow_) are associated with the decay of the water signal in the human brain.^[13]^ ADC_fast_ is associated with the intracellular volume in brain, whereas ADC_slow_ is associated with the extracellular volume in brain. Due to this biexponential behavior in brain, the use of DWI based on a monoexponential model can lead to inaccurate conclusions.^[22]^ Our study showed that ADC, a parameter of the monoexponential model, was increased in patients with type 2 diabetes as compared with nondiabetic controls, suggesting vasogenic brain edema in type 2 diabetes. However, it is not know whether this increase of ADC/vasogenic brain edema is due to the change in extracellular volume, intracellular volume, or both. In addition, the effect of cerebral perfusion on chronic brain damage cannot be evaluated using the monoexponetial model. In the biexponential model, we found that ADC_slow_ and a fraction of fast ADC (*f*) were increased and that ADC_fast_ was decreased in patients with type 2 diabetes as compared with nondiabetic controls. Normally, the intracellular volume is much more than the extracellular volume in the human brain. Type 2 diabetes can cause atherosclerosis and chronic ischemia, resulting in decreased cerebral perfusion. In addition, long-term uncontrolled diabetes can cause neurodegeneration and brain atrophy. Thus, the intracellular volume in the brain is decreased and the ratio of the extracellular volume to the intracellular volume is increased in patients with type 2 diabetes, leading to decreased ADC_fast_ and increased ADC_slow,_ respectively. Furthermore, ROC analysis showed that parameters of the biexponential model were better than the parameter of the ADC on the basis of the AUC, sensitivity, and specificity.

A stretched exponential model has been developed for continuous distribution of decay among sources.^[12]^ While the biexponential model of water diffusion assumes that there are at least 2 different diffusion rates, the stretched exponential model describes diffusion-related decay as a continuous distribution of sources decaying at different rates, without an assumption made about the number of participating sources. In this study, we found that there was no significance between parameters of stretched exponential model and parameters of biexponential model as indicated by the ROC analysis. However, we found that parameters of the stretched exponential model were superior to the parameter of the monoexponential model for the detection of chronic brain damage in type 2 diabetes patients. Our results showed that DDC was increased in patients with type 2 diabetes as compared with nondiabetic controls, whereas stretched exponential (α) was decreased. Diabetes-associated microvascular disease can cause chronic ischemia and hypoxia in the brain, resulting in chronic damage in the neuron, axon, and medullary sheath. These chronic brain damage can increase heterogeneity in diffusion, leading to increased DDC and decresed α. In addition, the DDC was significantly higher than the ADC, indicating that the DDC may be more accurate than the ADC to describe water diffusion in the brain.

Our study showed that DWI with multiple *b* values may be used for detection of chronic brain damage in patients with type 2 diabetes. Common manifestations of chronic brain damage in diabetes include lacunar infarction, leukoaraiosis, and brain atrophy, as shown in the MRI (+) group in our study. However, a large number of diabetes patients have no abnormalities on brain MRI. Considering this, we enrolled 2 groups of diabetes patients with or without abnormalities on brain MRI. Our results suggest that the centrum semiovale supplied by the ACA showed the most significant change in parameters, and the changes in the parameters ADC_slow_, *f*, and DDC were more significant than the changes of other parameters. These findings indicate that the centrum semiovale supplied by the ACA is vulnerable to chronic brain damage, which may be due to lack of collateral circulation and susceptibility to ischemia in this area. In addition, we found that ADC_slow_ had the highest performance for the detection of chronic brain damage, suggesting that ADC_slow_ might serve as a marker of monitoring type 2 diabetes patients.

Our study has several limitations. First, the number of subjects included in the study was relatively small. Second, counting within an ROI was a relatively subjective method for image analysis. Although we drew a small ROI to avoid the influence of blood vessels, cerebrospinal fluid, and infarction, we cannot exclude the influence of a partial volume effect. Third, we did not know whether medications affected the brain in enrolled patients.

In conclusion, our study shows that DWI can quantitatively access chronic brain damage in type 2 diabetes patients. Parameters of biexponential and stretched exponential models are superior to the parameter of monoexponential model. The ADC_slow_ of the centrum semiovale supplied by the ACA may be the best marker for the detection and monitoring of chronic brain damage in diabetes patients.
